# Quand la biopsie cutanée peut étiqueter une épilepsie

**Published:** 2011-10-22

**Authors:** Taoufiq Harmouch, Salim Gallouj, Kaoutar Znati, Aicha Slassi Sennou, Faouzi Belahcen, Afaf Amarti

**Affiliations:** 1 Laboratoire d'Anatomie Pathologique, CHU Hassan II, Fès, Maroc; 2 Service de Dermatologie, CHU Hassan II. Fès, Maroc; 3 Service de Neurologie, CHU Hassan II, Fès, Maroc

**Keywords:** Lafora, épilepsie, biopsie cutanée, Maroc

## Abstract

La maladie de Lafora (ML) représente une forme rare et grave d’épilepsie myoclonique progressive. C'est une affection à transmission autosomique récessive, hétérogène sur le plan génétique. Nous rapportons le cas d'une adolescente de 16 ans, issue de parents consanguins de premier degré, qui présente depuis l’âge de 14 ans des crises d’épilepsie et des myoclonies. L'examen neurologique a montré un syndrome cérébelleux et une détérioration intellectuelle. La biopsie cutanée était indispensable pour orienter le diagnostic. La ML a un pronostic constamment fatal. L’étude histologique confirme le diagnostic et l’étude moléculaire peut aider à établir un conseil génétique.

## Introduction

La maladie de Lafora est une maladie génétique autosomique récessive rare qui se manifeste par des épilepsies myocloniques progressives (EMP). Le diagnostic est évoqué devant l'association chez un adolescent d'une épilepsie avec des myoclonies et la détérioration cognitive progressive. Les anomalies à l’électroencéphalogramme sont caractéristiques et la confirmation est apportée par l'examen histologique par la présence de corps de Lafora au niveau des glandes sudoripares axillaires [1]. L’évolution est souvent fatale. Nous rapportons le cas d'une adolescente de 16 ans, issue de parents consanguins de premier degré, qui présente depuis l’âge de 14 ans des crises d’épilepsie et des myoclonies.

## Observation

**Figure 1:**
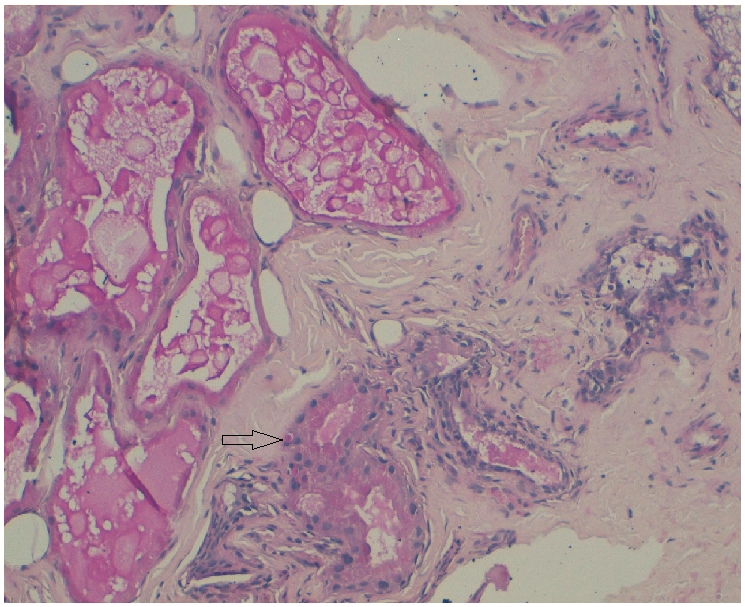
Corps de Lafora intracytoplasmiques (flèche) au niveau des cellules épithéliales bordant les canaux excréteurs des glandes sudoripares apocrines du creux axillaire. (GX 40, coloration par l'acide périodique de Schiff: PAS)

Patiente de 16 ans issue de parents consanguins de premier degré. Deux sœurs décédées par la même symptomatologie. Le début remonte à l’âge de 14 ans par l'apparition de crises généralisées tonico-cloniques et myocloniques dans un contexte de détérioration cognitive progressive. L'examen neurologique retrouve Syndrome cérébelleux cinétique. L’électroencéphalogramme (EEG) est en faveur d'une encéphalopathie épileptique. L’étude du LCR et le reste du bilan biologique sont normaux. L'imagerie par résonance magnétique (IRM) cérébrale a montré une Atrophie cérébrale. L’étude anatomopathologique de la biopsie cutanée effectuée au niveau de la région axillaire montre la présence de corps de Lafora au niveau des cellules épithéliales des glandes sudoripares apocrines. Ces corps de Lafora sont mieux visibles sur des coupes histologiques colorées par l'acide périodique de Schiff (PAS) (Figure 1). Ces données histologiques ont permis de confirmer le diagnostic de maladie de Lafora.

## Discussion

La maladie de Lafora (ML) est une forme particulièrement grave de l'EMP. La ML étant ubiquitaire, mais plus fréquente dans le pourtour méditerranéen [1]. Ses premières manifestations surviennent à l'adolescence: crises généralisées tonico-clonique ou clono-tonico-cloniques, myoclonus d'action et de repos, myoclonies négatives, mais aussi des crises partielles occipitales avec amaurose transitoire [2]. La ML est une maladie génétique autosomique récessive. Les études génétiques ont mis en évidence des variantes cliniques de la ML, et leur spectre devrait s'agrandir au fur et à mesure des progrès de l’élucidation des mécanismes génétiques. Il a par exemple été montré que les mutations dans l'exon 1 du gène EPM2A pouvaient produire un phénotype différent, avec début dans l'enfance parfois par des difficultés d'apprentissage, qui ne sont suivies que plus tard par l’évolution plus classique de la maladie [3]. Cette notion d'intervalle libre représente un élément important du diagnostic, comme cela fut le cas pour notre patiente. De même, le gène EMP2B semble être associé à une évolution un peu plus longue, et un peu moins sévère, de la ML et ceci indépendamment du type de mutation ou microdélétion constaté [4].

L’électroencéphalogramme (EEG), dont les modifications peuvent précéder l'installation des symptômes, met initialement en évidence une activité de fond normal e, parfois ralentie. Dans la moitié des cas il existe une activité diffuse de polypoints-ondes qui sont sporadiques ou en salves, spontanées ou provoquées par un mouvement ou par la marche, associées à un ralentissement progressif du rythme de fond et qui se majorent au fil du temps [1]. L'EEG réalisé à notre patiente a retrouvé des signes en faveur d'une encéphalopathie épileptique. A coté de la présentation typique de la maladie, qui s'applique à la grande majorité des cas, il existe des variantes notables qui nécessitent une confirmation histologique ou moléculaire.

Le rôle de la biopsie cutanée prélevée au niveau du creux axillaire est de confirmer le diagnostic de la ML en mettant en évidence des corps de Lafora ou de polyglucosan dans le cytoplasme des cellules épithéliales qui bordent les canaux excréteurs des glandes sudoripares apocrines. Ces inclusions PAS positifs caractéristique de la la maladie de Lafora sont présentes dans plusieurs organes comme le cerveau, le cœur, le foie et le muscle strié squelettique [2]. Les corps de Lafora sont des polyglycosans denses et phosphorylés, qui ressemblent à ceux des corps amylacés normaux constatés dans le cerveau des personnes âgées, mais leur localisation dans le péricaryon du neurone et dans les dendrites est caractéristique de la ML, car les polyglycosans physiologiques sont constatés le plus souvent au niveau des axones et des cellules gliales. La présence des corps de Lafora dans la biopsie axillaire chez des sujets jeunes est pathognomonique de la ML. Il faut cependant savoir que ces anomalies caractéristiques peuvent échapper à un œil peu exercé, et qu'une seconde lecture, ou une nouvelle biopsie axillaire, peuvent être nécessaires [2,5].

Les diagnostics différentiels sont discutés en fonction des du stade évolutif de la maladie. A un stade de début une épilepsie myoclonique juvénile ou autres formes idiopathiques sont évoqués. A la phase d’état le principal diagnostic différentiel est celui de la maladie d'Unverricht-Lundborg, alors qu’à la phase tardive de la ML toutes les étiologies d'EMP sont envisageables [6].

En l'absence de traitement curatif, la pharmaco-résistance et le ralentissement psychomoteur sont des facteurs limitant, et le décès survient 2 à 10 ans après le début de la symptomatologie, d'un mal épileptique et dans un état cachectique [1,4].

## Conclusion

La maladie de Lafora appartient au groupe des épilepsies myocloniques progressives. Toutefois, elle possède des caractéristiques cliniques et évolutives notables. La résistance aux antiépileptiques et la détérioration cognitive progressive doivent orienter le clinicien vers la réalisation d'une biopsie cutanée axillaire à la recherche de corps de Lafora caractéristique de la maladie. Une étude moléculaire à la recherche de mutations géniques peut étayer le diagnostic et servir de base pour un conseil génétique. Son pronostic est constamment fatal.
